# Orofacial Features, Oral Health-Related Quality of Life, and Exposure to Bullying in Osteogenesis Imperfecta: A Cross-Sectional Study

**DOI:** 10.3390/children11080900

**Published:** 2024-07-26

**Authors:** Alice Broutin, Jean-Pierre Salles, Valérie Porquet-Bordes, Thomas Edouard, Frédéric Vaysse, Emmanuelle Noirrit-Esclassan

**Affiliations:** 1 Paediatric Dentistry, University Toulouse III, CHU Toulouse, Reference Centre for Rare Oral Diseases, Centre for Anthropobiology & Genomics of Toulouse (CAGT) CNRS UMR 5288, 31400 Toulouse, Francefrederic.vaysse@univ-tlse3.fr (F.V.); 2 Endocrine, Bone Diseases and Genetics Unit, Reference Centre for Rare Diseases of Calcium and Phosphate Metabolism, OSCAR Network, ERN BOND, Children’s Hospital, Toulouse University Hospital, 31300 Toulouse, France; salles.jp@chu-toulouse.fr (J.-P.S.); porquet-bordes.v@chu-toulouse.fr (V.P.-B.); edouard.t@chu-toulouse.fr (T.E.); 3 Paediatric Dentistry, University Toulouse III, CHU Toulouse, Reference Centre for Rare Oral Diseases, ADES UMR 7268 CNRS EFS, 13005 Marseille, France

**Keywords:** osteogenesis imperfecta, bullying, malocclusion, oral health-related quality of life

## Abstract

Background/Objectives: Osteogenesis imperfecta (OI) is a rare genetic disease that is responsible for bone fragility, but also for dental malocclusions and dentinogenesis imperfecta (DI). The aim of this study was to assess whether the severity of dental malocclusion influenced the oral health-related quality of life (OHRQoL) and exposure to bullying in a paediatric OI population compared with a control group. Methods: Dental and occlusal characteristics were noted during oral and radiographic examination. The severity of malocclusion was assessed using the PAR index. P-CPQ, COHIP(34), and BCS-A questionnaires were used to evaluate, respectively, externally and self-perceived OHRQoL and bullying. Results: We included 39 patients with a mean age of 11.3 (±4.8 SD) in the OI group, and 45 patients with a mean age of 12.3 (±3.2 SD) in the control group. There were no significant differences between the two groups in terms of occlusal vertical and transverse dimensions. Patients with severe OI, presenting with bone fractures, bones deformities, and short stature, had significantly more anterior (*p* < 0.05) and posterior openbites (*p* < 0.05) and more DI (*p* < 0.05) compared to patients who had moderate or mild OI. Self-perceived OHRQoL was negatively impacted by the disease (*p* = 0.01), particularly in the domains of oral health (*p* < 0.05) and self-image (*p* < 0.001), but not by its severity. Exposure to bullying did not differ significantly between the two groups, although more patients with OI reported being teased (21.4% face to face and 7.1% online vs. 14.6% and 2.4% in the control group). Conclusion: Interventions for dental malocclusion and oral health in OI patients would help to improve their quality of life and self-image.

## 1. Introduction

Osteogenesis imperfecta (OI) is a rare genetic bone disease (occurring in 1 in 10,000 to 20,000 births) [1]. It is responsible for bone fragility, leading to numerous fractures, and even bone and spine deformities and short stature. The severity and frequency of these issues vary depending on the type of OI. The disease is phenotypically and genetically heterogeneous. It has been demonstrated that these symptoms have a negative impact on patients’ quality of life [2,3]. To date, there is no cure for OI [3]. Multimodal treatments, including anti-resorptive treatments, surgical intervention, physical therapy, and psychosocial support, help to manage and treat pain, limiting disabilities and improving the patients’ quality of life [4].

However, these disabling skeletal manifestations are not the only potential sources of discomfort, pain and stigmatization. Patients with OI often exhibit a characteristic triangular face with a broad forehead, hypoplasia of the mid face, and a prognathic mandible. Dentinogenesis imperfecta (DI) and dental malocclusions, mainly Angle Class III malocclusion, anterior and/or posterior crossbites, and/or open bites, are commonly seen in patients with OI [5,6]. Dental malocclusions tend to worsen with age and are likely to cause functional difficulties when chewing and eating, as well as issues with dental aesthetic.

Thus, it seems relevant to focus on the oral health-related quality of life (OHRQoL) of patients with OI. OHRQoL is defined as “a multidimensional construct that includes a subjective evaluation of the individual’s oral health, functional well-being, emotional well-being, expectations and satisfaction with care, and sense of self” [7]. It is assumed that a patient’s OHRQoL will have an impact on their general quality of life. Many studies report that malocclusions have negative effects on the OHRQoL of children and adolescents [8,9]. On the other hand, a relationship has been highlighted between malocclusion or structural defects and exposure to bullying among young adolescents [10]. Given their orofacial symptoms, it seems useful to evaluate this exposure in patients with OI.

Considering the importance, multiplicity, and variability of the craniofacial and oral repercussions of the disease, it seems relevant to assess OHRQoL and exposure to bullying in patients with OI, and the correlation of these factors with dental and occlusal characteristics. This is a necessary step towards improved management in dental and orthodontic care. Of particular importance is adapting the therapeutic objectives to the specific needs of the patient. In the present study, we evaluate dental and occlusal characteristics, the severity of dental malocclusion, OHRQoL, exposure to bullying, and the correlations of these factors in an OI population and a control group.

## 2. Materials and Methods

### 2.1. Ethical Approval

This study, falling outside the Jardé laws and registered under number ID-RCB 2021-A02561-40, obtained launch authorization on 29 October 2021.

Every patient and parent or legal representative were advised about our research by the principal investigator and received an information form about this study’s setting, its non-invasive nature, and its aims. Each of them declared that they had no objection to participating in this study.

### 2.2. Recruitment and Setting

This non-interventional cross-sectional observational study was conducted at the French University Hospital Centre, Toulouse, from November 2021 to May 2023. Patients were recruited during their OI or dental check-ups. Forty-five of the OI patients of the Toulouse Reference Centres for Rare Oral Diseases or for Constitutional Bone Diseases met the inclusion criteria. For a confidence interval of 90%, a margin of error of 5%, and a response distribution of 50%, the required sample size included 39 responses from a population of 45. Each patient with OI was asked to participate in this study. Control group patients treated in private practice were included during dental follow-up consultations.

Oral examination was carried out. This was combined with an orthopantomogram for children over 6 years old. If the orthopantomogram was less than 3 years old, no new examination was performed. Parents and patients over 8 years old were asked to answer questionnaires evaluating OHRQoL and bullying.

### 2.3. Participants

- Inclusion criteria

OI group:

- Patients diagnosed with osteogenesis imperfecta (based on genetic and/or skeletal manifestations);
- Seen at the Toulouse Reference Centres for Rare Oral Diseases or for Constitutional Bone Diseases for care or follow-up consultation;
- Aged 3 to 19.

Control group:

- Healthy patients consulting for dental follow-up in 2 private practices;
- Aged 3 to 19.

In both groups, patients had to be covered by their own healthcare insurance or that of their parents. Each patient and at least one of their parents (or legal representatives) was required to understand French.

- Non-inclusion criteria

- Patients attending bone disease diagnostic consultations;
- Patients who were unable to read questionnaires or for whom neither of their parents (or legal representatives) were able to read questionnaires.

- Exclusion criteria

Missing data.

### 2.4. Data Collection

#### 2.4.1. Severity of the OI

Severity (mild, moderately severe, or severe) was determined by the medical team of the paediatric bone disease centre in compliance with the National Diagnosis and Care Protocol [11] and the Sillence classification [12]. Patients with severe OI presented with bones fractures from the perinatal period, bones deformities, and short stature, whereas patients with mild OI essentially presented with fractures from walking age following minor trauma, normal or near-normal growth velocity and height, and an annualized fracture rate of less than or equal to 1. Patients with the moderately severe form presented an intermediate clinical picture, occasionally displaying congenital fractures, decreased growth velocity and height, and an anterior bowing of the legs and thighs.

#### 2.4.2. Dental and Occlusal Characteristics

These were noted during oral and radiographic examination by two paediatric dentists (AB and EN).

- Dental characteristics

- Dentinogenesis imperfecta was diagnosed based on clinical features and radiographic findings. Clinical signs included soft, blue-to-brown translucent discoloured teeth; significant spontaneous attrition; and premature tooth loss. Radiography results presented with poor mineralization density, bulbous crowns, cervical constriction, short roots, and pulp canal obliteration [13].
- The history of caries was assessed both clinically and radiographically. We considered a patient to present a history of caries when at least one tooth had a cavity or a filling.
- Each participant and their parents (or legal representative) were asked if there was a history of orthodontic treatment (ongoing or already finished).

- Occlusal characteristics

- Sagittal dimension. Canine Angle classification was used to define the canine relationship as Class I, II, or III. A half-unit Class II or a half-unit Class III was considered to be Class II or Class III, respectively.

Overjet was defined as the distance from the most labial point of the incisal edge of the maxillary incisors to the most labial surface of the corresponding mandibular incisors. It was measured to the nearest half millimeter, parallel to the occlusal plane [14]. An overjet greater than 3 mm was considered to be an increased overjet [15].

- Vertical dimension. Overbite was considered to be the vertical overlap of the incisors when the posterior teeth were in contact. The normal range was determined to be one-third coverage of the upper and lower incisors [14]. An overbite greater than one-third coverage of the upper and lower incisors was considered to be an increased overbite.

An anterior open bite was recorded if the upper and lower incisors were vertically separated. A posterior open bite was recorded if one or more of the upper and lower molars and premolars were vertically separated by more than 2 mm, except during eruption.

- Transverse dimension. A posterior crossbite was recorded when the buccal cusps of the maxillary premolars and/or molars occluded lingually to the buccal cusps of the mandibular antagonists (at least one pair of teeth, uni- or bilateral). Teeth in an edge-to edge position were also included [14].

#### 2.4.3. Malocclusion

The Peer Assessment Rate (PAR) index [15] was used to assess the severity of malocclusion. A score and a weighting coefficient were assigned to the various occlusal features. These were likely, due to their deviation from the standard, to constitute malocclusions. The values were then added together to obtain the PAR score, representative of the degree of severity of the malocclusion: a score of 0 corresponds to perfect intra- and interarch relationships. The higher the score (rarely above 50), the more severe the malocclusion.

#### 2.4.4. OHRQoL

To evaluate patients’ OHRQoL, parents answered the 33 questions of the Parental–Caregivers Perceptions Questionnaire (P-CPQ) [16] (Supplementary Materials), and children older than 8 answered the 34 questions of the Child Oral Health Impact Profile (34) questionnaire (COHIP(34)) [17]. Answers to these questions were scored, and OHRQoL was evaluated based on the total score obtained for each questionnaire.

The P-CPQ began with two questions asking parents for a global rating of their child’s oral health. The P-CPQ is composed of 33 items. These are divided into four sub-scales, including the following: orals (OS—6 items), functional limitations (FL—8 items), emotional well-being (EWB—9 items), and social well-being (SWB—10 items). The questions referred to the frequency of events that occurring during the previous 3 months. A five-point Likert-type scale was used, with the following options for responses: “Never” (score 0), “Once or twice” (1), “Sometimes” (2), “Often” (3), and “Nearly every day” (4) [16].

The COHIP(34) questionnaire consists of 34 items, with a five-point Likert-type scale covering several theoretical domains: (i). “Oral health”: ten negatively worded questions are used to evaluate specific oral symptoms (e.g., pain, spots on teeth). (ii). “Functional well-being”: six questions relate to the child’s ability to carry out specific everyday tasks or activities (e.g., speaking clearly, chewing). (iii). “Social-emotional well-being”: this uses eight negatively worded items concerning peer interactions and mood states. (iv) “School environment”: this uses four negatively worded items to evaluate tasks associated with the school environment. (v) “Self-image”: this includes six questions addressing experience (e.g., been confident, felt attractive) and feelings. (vi) Tere is one question assessing perceptions of general health.

#### 2.4.5. Bullying

Finally, the Bullying and Cyberbullying Scale for Adolescents questionnaire (BCS-A) [18] was completed by patients over 8 years old in order to evaluate bullying and cyberbullying.

The BCS-A comprises 26 items and two subscales: a Victimization Scale (13 items) and a Perpetration Scale (13 items). Items are scored from 0 (never) to 4 (four times or more) and the scoring range is 0 to 104. High scores indicate higher levels of bullying [18]. A young person was considered to be a bully or a victim of bullying if he or she answered “three or more” at least once on the “bullied/er face to face” or “bullied/er via social media” questionnaire.

### 2.5. Statistical Analysis

Descriptive statistics were used to analyse the data collected through examinations and questionnaires. To compare patients with each other, non-parametric Pearson’s chi-squared tests, Fisher’s exact tests, and Wilcoxon rank-sum tests were used. Spearman’s correlation coefficient was calculated to evaluate the relationship between malocclusion severity and OHRQoL. To assess the relationship between OI severity and PAR index values, we used a Kruskal–Wallis rank-sum test.

The data were analysed using R^®^ software (Bell, Murray Hill, NY, USA, version 4.4.1 (2024-06-14)). The threshold of significance was fixed at 0.05.

## 3. Results

During the inclusion period, 42 patients with OI were included. Three of them were excluded because of missing data. In the control group, 45 patients were included. The mean age was 11.3 (±4.8 SD) for the OI group and 12.3 (±3.2 SD) for the control group. 

Table 1 shows the demographic data of participants included in both the OI and control groups. Overall, 95% of the patients with OI had a history of intra-venous bisphosphonate therapy.

The results of oral examinations are set out in Table 2 and Table 3. Patients with OI presented with significantly more canine Angle Class III occlusion (46% vs. 13% in the control group; *p* < 0.01). Patients with severe OI presented with significantly more anterior (27% vs. 0% in mild OI form; *p* < 0.05) and posterior open bites (36% vs. 0% in moderately severe form and 5% in mild form; *p* < 0.05) and DI (64% vs. 14% in moderately severe and 24% in mild form; *p* < 0.05), while increased overjets were seen only in patients with the moderately severe form of the disease (43% vs. 0% in severe and mild forms; *p* < 0.01).

**Figure 1:**
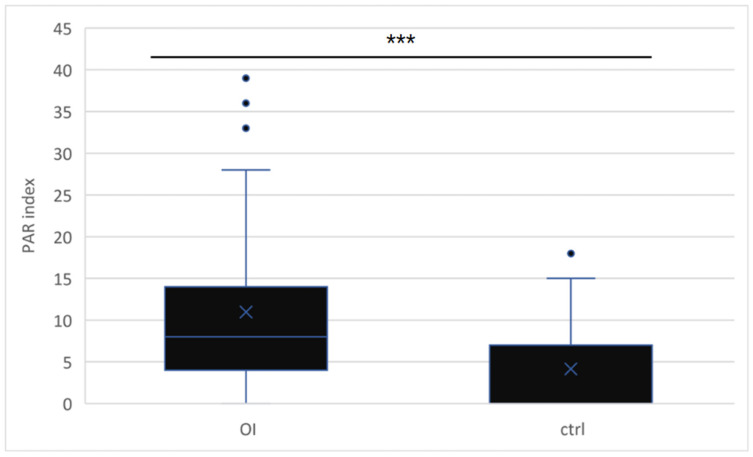
PAR index in the OI (11, SD 9.7) vs. control group (4, SD 5.8) (OI: osteogenesis imperfecta group/ctrl: control group) (***: *p* < 0.001).

**Figure 2:**
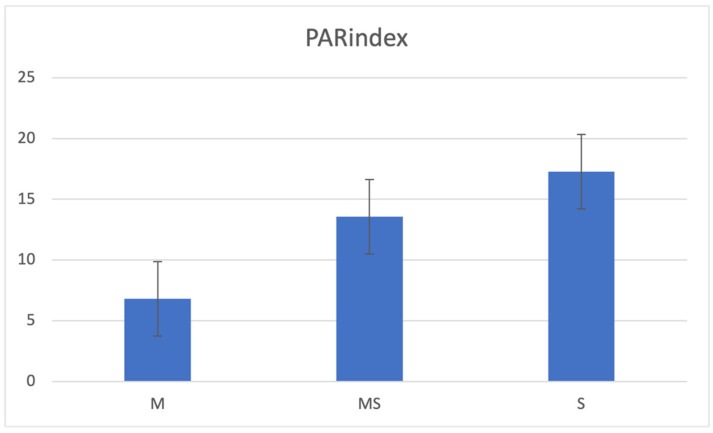
PAR index in the mild (M: 6.8, SE 0.9), moderately severe (MS: 13.6, SE 3.7) and severe (S: 17.3, SE 3.9) OI groups: the difference among the three groups is significant (*p* < 0,05, Kruskal–Wallis rank-sum test).

According to the PAR index assessment (Figure 1 and Figure 2), dental malocclusion was more severe in the OI group (11, SD 9.7) than in the control group (4, SD 5.8; *p* < 0.001), and the severity of malocclusion varied with OI severity (mild: 6.8, SE 0.9; moderately severe: 13.6, SE 3.7; severe: 17.3, SE 3.9; *p* < 0.05).

**Figure 3:**
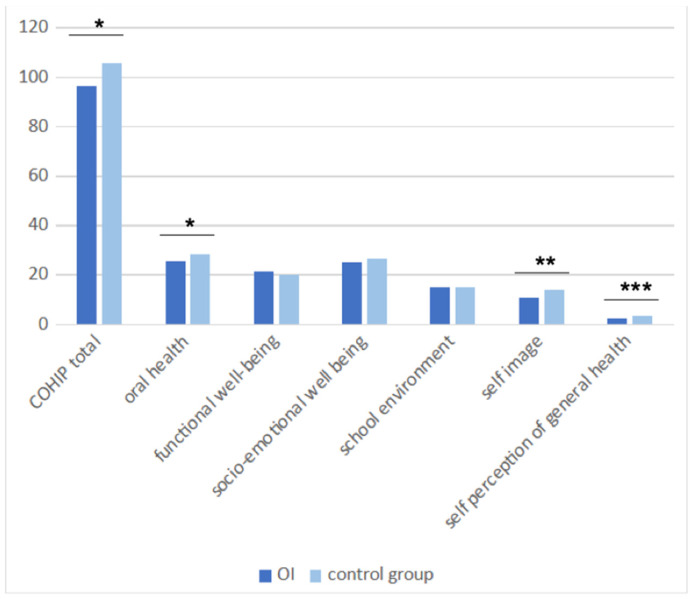
Self-perceived OHRQoL measured using COHIP(34) among the children older than 8. (OHRQoL: oral health-related quality of life/COHIP(34): Child Oral Health Impact Profile (34)) (*: *p* < 0.05/**: *p* < 0.01/***: *p* < 0.001).

Self-perceived OHRQoL, assessed with COHIP(34) (Figure 3), was significantly impacted in the OI group (96.4 vs. 105.8 in the control group; *p* = 0.01), particularly in the domains of oral health (25.6 vs. 28.5 in the control group; *p* < 0.05) and self-image (10.7 vs. 14.1 in the control group; *p* < 0.01). At the same time, the self-perception of general health was significantly lower in the OI group (2.5 vs. 3.3 in the control group; *p* < 0.001).

**Figure 4:**
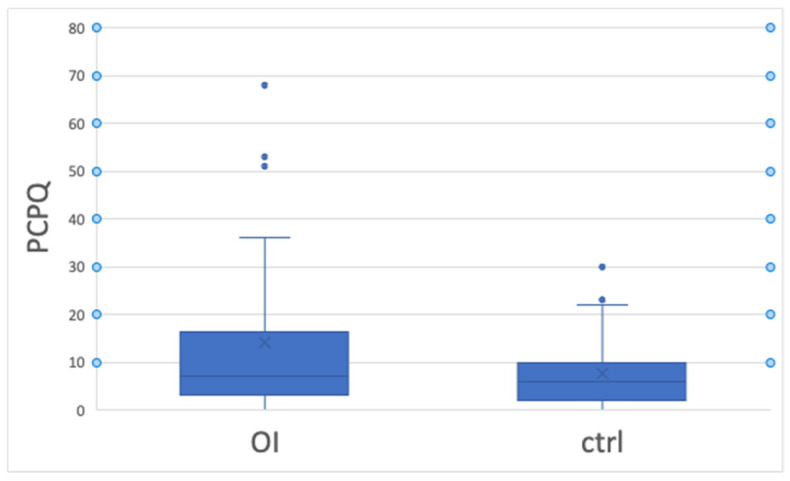
OHRQoL externally perceived by P-CPQ. OHRQoL: oral health-related quality of life/P-CPQ: Parental–Caregivers Perceptions Questionnaire.

However, externally perceived OHRQoL—evaluated with the P-CPQ (Figure 4)—was not significantly impacted by the OI, and no self- or externally perceived OHRQoL reading was significantly influenced by the severity of the disease. 

The presence of DI did not significantly impact OHRQoL (either self- or externally assessed).

There was no significant association between OI and exposure to bullying, although more patients in the OI group reported being exposed (21.4% and 7.1% online) than those in the control group (14.6% and 2.4% online) (Table 4).

Scores for the history of caries, dental appearance, and tooth sensitivity did not differ significantly between the patients of the two groups (Table 2 and Table 4).

No significant correlation could be demonstrated between malocclusion severity and OHRQoL, irrespective of whether it was self- or externally assessed. 

## 4. Discussion

In the present study, patients with OI had more frequent malocclusions, with a correlation between the severity of malocclusions and that of the OI. On the other hand, OHRQoL was negatively impacted by the disease, but this was not correlated with the severity of the OI.

Malocclusions, evaluated using the PAR index, were significantly more severe in the OI group than those in the control group. Before our study, Rizkallah et al. [19] came to the same conclusion using the same index. However, their reported scores were quite different from ours. In their population of 49 patients with OI, the average PAR score was 31.1, compared to 11 in our study. This difference may be due to the predominance of severe forms of OI in their study population (only 16% of patients with mild OI), compared to the predominance of the mild form in ours (54%), given that the ages and exposure to bisphosphonate therapy were similar in both populations. Moreover, their patients were seeking orthodontic treatment, while 36% of our OI group patients had a history of orthodontic treatment (ongoing or already finished). However, it was noticeable in our study that the more severe the form of OI, the greater the severity of the malocclusions and the more heterogeneous they were.

The PAR index was developed to assess the severity of malocclusion and the success of orthodontic treatments by comparing pre- and post-treatment scores [15]. Several other studies have arrived at similar results to ours using other indices, such as Dental Health Component of the Index of Orthodontic Treatment Need (DHC-IOTN), the Dental Aesthetic Index (DAI) [20], and the American Board of Orthodontics (ABO) Discrepancy Index [21]. The Index of Complexity Outcome and Need ICON could also have been used [22].

We did not find any other study assessing the PAR index score in a population specifically affected by OI. However, a German study looked at changes to this malocclusion score before and after orthodontic treatment in patients with special health care needs (suffering from syndromes with craniofacial repercussions) [23]. The children in their study group had significantly higher pre- and post-treatment PAR scores (respectively, 21 and 6) than their control group (17 and 0). Given that more than one-third of our OI group patients and almost half of our control group patients had a history of orthodontic treatment, we take the view that our PAR index scores are consistent with these findings. Thus, with early orthodontic treatment, the occlusal impact of OI can be substantially reduced.

It is noteworthy that canine Angle Class III, associated in most cases with anterior crossbite, was significantly more frequent (*p* < 0.05) in our OI group than in the control group (47% vs. 13%). Previously, Rizkallah et al. [19] had reported Angle Class III in 57% of patients in their OI group, compared to 62.5% in the study by Chang et al. [6] and 73.1% in that by Nguyen et al. [20]. The literature review by Prado et al. [5], focusing on malocclusions in patients with OI, confirmed that Angle Class III malocclusion and anterior crossbite occur at higher rates in OI individuals compared to those without OI. These malocclusions in patients with OI are linked not only to mandibular prognathism, but also to maxillary retrognathism and hypoplasia, caused by a primary growth defect of the cranial base [5,6,24]. According to Waltimo-Sirén et al. [24], both maxillary and mandibular growth may be deficient in OI patients, and so reversed overjet may result from disproportionate sagittal growth of the jaws, with maxillary growth more deficient than that of the mandible. In the present study, no cephalometric examination was used to assess skeletal malocclusion. It is therefore possible that we underestimated the orthodontic pathologies of these patients.

Significantly more anterior and posterior open bites were observed in our patients with severe OI, as in the 2013 study by Rizkallah et al. [19]. Furthermore, in our study population, the severity of malocclusion increased with the severity of OI, which was in agreement with the results obtained by Jensen and Lund [25], Waltimo-Sirén et al. [24], and Rizkallah et al. [19]. Given that malocclusions became more dominant with increased age in these patients [6], and that considerably greater forces and longer time intervals should be scheduled for each orthodontic treatment [26], both because of both OI and anti-resorptive treatments, we conclude that early diagnosis and interceptive orthodontic therapies should be encouraged for all patients with OI, and particularly for those with a severe form. If intervention is not arranged when the individual is still growing, the risk that orthognathic surgery will be required in adulthood to correct malocclusion is much higher [5]. Although such interventions have been reported in the literature, surgical complications have been described and these patients should be managed very carefully [26]. To date, no case of osteonecrosis of the jaw has been reported in patients treated with bisphosphonates for their OI, and bisphosphonate medication seems to have no significant impact on healing after oral surgery in this population [26]. Despite their pathology, 36% of patients in our OI group had access to orthodontic treatment. This is somewhat lower than the control group (43%), but the difference can be explained by their mean ages, which were 11.3 in the OI group and 13.1 in the control group.

One-third of the OI population included in our study presented with DI. This was in line with previous studies, which reported that 24.7% to 47.1% of OI patients presented with DI [13,27,28,29,30]. We also found a significant association between the severity of the disease and the presence of DI, which was consistent with the conclusions of previous studies [13,28,30,31]. All studies found in the literature, with one exception, based themselves on both clinical and radiological criteria in order to diagnose the presence of DI, like our method [13,27,28,29,30]. Marçal et al. [31] only used radiological criteria. Some authors were interested in the histological structure of dentin in patients with OI and highlighted subclinical histological manifestations of DI in patients with OI without clinical or radiographic signs of DI [32,33]. Therefore, the prevalence of DI in patients with OI may be underestimated in clinical studies, including ours.

Twenty-two studies have stressed the influence of malocclusion and structural defects on the risk of exposure to bullying among young adolescents [10]. Medically fragile young people such as patients with OI may be at risk of bullying by their peers because of their bone deformities, along with short stature, DI, activity-limiting physical characteristics, and frequent school absences for repeated hospitalisation [34]. Surprisingly, our study did not find any significant difference in exposure to peer victimization between young people with and without OI, although they were more frequently teased. This was confirmed with the scores in the domains of social well-being and the school environment, which were evaluated with the COHIP(34) questionnaire. These did not differ from those of the healthy group. Successful social adjustment and average to above-average cognitive abilities may protect them from peer victimization [35]. Moreover, as their OI symptoms are present from a very young age, children are aware of their condition early on. Specifically, their self-perception of general health was significantly lower in our OI group. Therefore, they may become more resilient and develop adaptive social skills to overcome their physical difficulties [36].

Previous studies showed that malocclusions have a negative impact on OHRQoL, especially from 11 years old [9]. Severe malocclusions in the aesthetic area have an impact on OHRQoL in children and adolescents, predominantly in the dimensions of emotional and social wellbeing [8]. Blanch et al. [37] even suggested considering malocclusion as a disability.

Our results indicate that OHRQoL is negatively impacted by OI, particularly in the domains of oral health and self-image. This could be related to DI and the malocclusions caused by the disease. However, contrary to previous studies, which highlighted the impact of the severity of OI [38,39,40] or the type of malocclusion [41] on OHRQoL, none of these parameters had a significant influence in our population. Although it is not clear which factors carried more weight in predicting lower OHRQoL, all of these conclusions indicated the importance of managing the oral repercussions of the disease in order to improve the OHRQoL.

In previous studies, OHRQoL has been assessed in OI patients using various scales: namely, researchers used the “8 to 10 years” and “11 to 14 years” age-specific versions of the Child Perceptions Questionnaire (CPQ) [38,41,42], and the COHIP-SF (the short form of the COHIP, consisting of 19 questions) [39] or OHIP (adult version, composed of 49 questions) [40]. COHIP(34) was not used in any of the other five articles but we in fact used it, impairing comparability. However, Broder et al. used it to assess OHRQoL in 4 groups: patients seeking paediatric dentistry treatment, orthodontic treatment, and craniofacial care, and a control group of children not seeking dental treatment [43]. Compared with these groups, our OI group presented a mean COHIP(34) score (96) higher than that of Broder et al.’s craniofacial group (87.1), but similar to their paediatric (97.7) and orthodontic groups (97.2). The control group of Broder et al. gave similar results to our own (respectively, 102.3 and 105). Thus, our group of OI patients has an OHRQoL closer to that of a patient seeking orthodontic treatment than to that of a patient seeking craniofacial care. It would be interesting to compare the PAR scores of our patients with those of patients seeking orthodontic treatment or craniofacial care in order to clarify the impact of the severity of the malocclusion on the one hand, and the perception of the disease and its consequences on the other hand.

When it comes to the external evaluation of OHRQoL, parents of young people with OI did not report poorer OHRQoL than their children. We hypothesise that they pay more attention to the other symptoms of the disease.

Our study evaluated the influence of OI on oral condition and OHRQoL for 39 children and adolescents with OI, which may appear to be a small cohort. We would note, however, that OI is a rare disease and that the size of our cohort seems consistent with those found in the literature. In our survey of studies assessing the oral condition of children with OI, we found cohorts of 40 children in the article of O’Connell and Marini [44], and cohorts of 68 children in the articles of Malmgren and Norgren [28] and Nguyen et al. [13]. As for monocentric studies evaluating OHRQoL in OI populations, Pantoja et al. [42] included 30 children and adolescents with OI, Gjørup et al. [40] included 73 adults, and Cachia Mintoff et al. included [39] 106 children.

Also, the present study had the benefit of a control group. When considering dental characteristics, only Marçal et al. [31] compared their OI group of 24 patients with a control group consisting of 48 patients. As regards malocclusion, five of the six articles included in the systematic review by Prado et al. [5] had control groups to assess cephalometric data [6,24,25] or evaluate the severity of malocclusion [19] or the need for orthodontic treatment [20]. Finally, considering OHRQoL in OI patients, none of the five articles dealing with this subject included a control group made up of healthy patients [38,39,40,41,42]. Gjørup et al. [40] had a group of patients without OI, but with X-linked hypophosphataemia.

## 5. Conclusions

OI is a rare genetic disease which impacts not only long bones but also craniofacial bones and oral condition. The severity of the disease and its clinical manifestations can vary considerably from one patient to another. In our study, young people with OI presented more severe and frequent malocclusions, particularly canine Angle Class III, than a healthy age–gender-matched control group. The more severe the OI, the more frequent anterior and posterior open bites and DI were, and the more severe malocclusion became. OHRQoL was negatively impacted by the disease, but not by its severity. Therefore, early orthodontic and dental treatment would help to improve patients’ oral health, as well as their self-image. From a socio-emotional health perspective, those patients are not more exposed to bullying than the general population. The factors operating to protect against peer victimization should be more deeply explored in young people with OI.

## Figures and Tables

**Table 1:** Demographic data.

		OI Group		Control Group	
		N (%)	Mean Age	N (%)	Mean Age
Total		39	11.3 (±4.8 SD)	45	12.3 (±3.2 SD)
Female/male		18 (46)/21 (54)		24 (53)/21 (47)	
OI severity	Mild	21 (54)	11.1		
	Moderately severe	7 (18)	10.1		
	Severe	11 (28)	12.5		

**Table 2:** Oral examination: OI vs. control group.

			OI Group N (%)	Control Group N (%)	*p*-Value
Total			39	45	
Dentinogenesis imperfecta			13 (33)	0 (0)	<0.001
History of orthodontic treatment			14 (36)	18 (40)	- *^1^
History of caries			15 (38)	12 (27)	-
Occlusal sagittal dimension	Canine Class	Class 1	19 (49)	31 (69)	<0.01
		Class 2	2 (5)	8 (18)	
		Class 3	18 (46)	6 (13)	
	Increased overjet *^2^		3 (8)	4 (9)	-
Occlusal vertical dimension	Increased overbite *^3^		9 (23)	4 (9)	-
	Anterior open bite		5 (13)	5 (11.1)	-
	Posterior open bite		5 (13)	5 (11.1)	-
Occlusal transverse dimension	Posterior cross bite		6 (15)	3 (7)	-

*^1^ Non-significant. *^2^ Increased overjet: >3 mm. *^3^ Increased overbite: >1/3 coverage of the upper and lower incisors.

**Table 3:** Oral examination in mild, moderately severe, and severe OI.

			Mild OI N (%)	Moderately Severe OI N (%)	Severe OI N (%)	*p*-Value
Total			21 (54)	7 (18)	11 (28)	
Dentinogenesis imperfecta			5 (24)	1 (14)	7 (64)	<0.05
History of orthodontic treatment			9 (43)	2 (29)	3 (27)	- *^1^
History of caries			7 (33)	4 (57)	4 (36)	-
Occlusal sagittal dimension	Canine Class	Class 1	12 (57)	4 (57)	3 (27)	-
		Class 2	0 (0)	1 (14)	1 (9)	
		Class 3	9 (43)	2 (29)	7 (64)	
	Increased overjet *^2^		0 (0)	3 (43)	0 (0)	<0.01
Occlusal vertical dimension	Increased overbite *^3^		7 (33)	1 (14)	1 (9)	-
	Anterior open bite		0 (0)	2 (29)	3 (27)	<0.05
	Posterior open bite		1 (5)	0 (0)	4 (36)	<0.05
Occlusal transverse dimension	Posterior cross bite		2 (10)	1 (14)	3 (27)	-

*^1^ Non-significant. *^2^ Increased overjet: >3 mm. *^3^ Increased overbite: >1/3 coverage of the upper and lower incisors.

**Table 4:** Results from questionnaires among the children older than 8.

		OI group			Control Group	*p*-Value
		Mild OI	Moderately Severe OI	Severe OI		
Dental appearance		6.2			7.1	- *^2^
		6.3	6	6		-
Tooth sensitivity		4.1			3.7	-
		4.6	5	2.6		-
Bullying self-assessed with B-CSA *^1^	“Face to face” victim (%)	21.4%			14.6%	-
		20%	20%	25%		-
	Online victim (%)	7.1%			2.4%	-
		7%	0%	13%		-
	“Face to face” bully (%)	7.1%			7.3%	-
		13%	0%	0%		-
	Online bully (%)	0%			2.4%	-
		0%	0%	0%		

*^1^ B-CSA: Bullying and Cyberbullying Scale for Adolescents. *^2^ Non-significant.

## Data Availability

The datasets used and/or analysed during the current study are available from the corresponding author on reasonable request. The data are not publicly available due to legal reason.

## Supplementary Materials

The following supporting information can be downloaded at: https://www.mdpi.com/article/10.3390/children11080900/s1, File S1: PCPQ French English. File S2: COHIP. File S3: Bullying questionnaire.
