# Supplementary material for: Orofacial Features, Oral Health-Related Quality of Life, and Exposure to Bullying in Osteogenesis Imperfecta: A Cross-Sectional Study
Source: Children (Basel). 2024 Jul 26;11(8):900. doi: 10.3390/children11080900 (PMC11352506; doi:10.3390/children11080900)
Supplement: Supplementary file 1 [file children-11-00900-s001.zip › File S1. PCPQ french english.pdf]

Original version	French version
SECTION 1: Child’s oral health and wellbeing	SECTION 1: *La santé bucco-dentaire et le bien-être de l’enfant.*
a. How would you rate the health of your child’s teeth, lips, jaws and mouth?<br>☐Excellent<br>☐Very good<br>☐Good<br>☐Fair<br>☐Poor	a. *Comment évaluez-vous la santé des dents, lèvres, mâchoires ou bouche de votre enfant*<br>☐Excellente<br>☐Très bonne<br>☐Bonne<br>☐Moyenne<br>☐Mauvaise
b. How much is your child’s overall wellbeing affected by the condition of his/her teeth, lips, jaws or mouth?<br>☐Not at all<br>☐Very little<br>☐Some<br>☐A lot<br>☐Very much	b. *A quel point le bien-être de votre enfant est affecté par l’état de ses dents, lèvres, bouche ou mâchoires.*<br>☐Pas du tout<br>☐Très peu<br>☐ Un petit peu<br>☐ Beaucoup<br>☐ Enormément
**SECTION 2: The following questions ask about symptoms and discomfort that children may experience due to the condition of their teeth, lips, mouth and jaws!**	**SECTION 2 : *Les questions suivantes portent sur les symptômes et l’inconfort que les enfants peuvent ressentir à cause de l’état de leurs dents, lèvres, bouche ou mâchoires.***
During the last 3 months, how often has your child had:	*Au cours des trois derniers mois, combien de fois votre enfant a t-il eu:*
1. Pain in the teeth, lips, jaws or mouth?<br>☐Never<br>☐Once or twice<br>☐Sometimes<br>☐Often<br>☐Everyday or almost everyday	1. Mal aux dents, aux lèvres, aux mâchoires ou à la bouche?<br>☐Jamais<br>☐Une ou deux fois<br>☐Quelques fois<br>☐Souvent<br>☐ Tous les jours, ou presque tous les jours
2. Bleeding gums?<br>☐Never<br>☐Once or twice<br>☐Sometimes<br>☐Often<br>☐ Everyday or almost everyday	2. Les gencives qui saignent?<br>☐Jamais<br>☐Une ou deux fois<br>☐Quelques fois<br>☐Souvent<br>☐ Tous les jours, ou presque tous les jours
3. Sores in the mouth?<br>☐Never<br>☐Once or twice<br>☐Sometimes<br>☐Often<br>☐ Everyday or almost everyday	3. Des endroits douloureux dans la bouche?<br>☐Jamais<br>☐Une ou deux fois<br>☐Quelques fois<br>☐Souvent<br>☐ Tous les jours, ou presque tous les jours
4. Bad breath?<br>☐Never<br>☐Once or twice<br>☐Sometimes<br>☐Often<br>☐ Everyday or almost everyday	4. Une mauvaise haleine ?<br>☐Jamais<br>☐Une ou deux fois<br>☐Quelques fois<br>☐Souvent<br>☐ Tous les jours, ou presque tous les jours
5. Food stuck in the roof of the mouth?	5. De la nourriture collée au palais?

☐Never<br>☐Once or twice<br>☐Sometimes<br>☐Often<br>☐ Everyday or almost everyday	☐Jamais<br>☐Une ou deux fois<br>☐Quelques fois<br>☐Souvent<br>☐ Tous les jours, ou presque tous les jours
6. Food caught in or between the teeth?<br>☐Never<br>☐Once or twice<br>☐Sometimes<br>☐Often<br>☐ Everyday or almost everyday	6. De la nourriture coincée dans ou entre les dents?<br>☐Jamais<br>☐Une ou deux fois<br>☐Quelques fois<br>☐Souvent<br>☐ Tous les jours, ou presque tous les jours
7. Difficulty biting or chewing foods such as fresh apple, corn on the cob or<br>firm meat?<br>☐Never<br>☐Once or twice<br>☐Sometimes<br>☐Often<br>☐ Everyday or almost everyday	7. Des difficultés à croquer ou à mâcher de la nourriture comme une pomme ou un steak?<br>☐Jamais<br>☐Une ou deux fois<br>☐Quelques fois<br>☐Souvent<br>☐ Tous les jours, ou presque tous les jours
During the last 3 months, because of his/her teeth, lips, mouth, or jaws,<br>how often has your child:<br>8. Breathed through the mouth?<br>☐Never<br>☐Once or twice<br>☐Sometimes<br>☐Often<br>☐ Everyday or almost everyday	*Au cours des trois derniers mois, à cause de ses dents, lèvres, bouche ou mâchoires, combien de fois votre enfant a-t-il :*<br>8. Respirer par la bouche?<br>☐Jamais<br>☐Une ou deux fois<br>☐Quelques fois<br>☐Souvent<br>☐ Tous les jours, ou presque tous les jours
9. Had trouble sleeping?<br>☐Never<br>☐Once or twice<br>☐Sometimes<br>☐Often<br>☐ Everyday or almost everyday	9. Eu des difficultés à dormir?<br>☐Jamais<br>☐Une ou deux fois<br>☐Quelques fois<br>☐Souvent<br>☐ Tous les jours, ou presque tous les jours
10. Had difficulty saying any words?<br>☐Never<br>☐Once or twice<br>☐Sometimes<br>☐Often<br>☐ Everyday or almost everyday	10. Eu des difficultés à prononcer des mots quels qu'ils soient ?<br>☐Jamais<br>☐Une ou deux fois<br>☐Quelques fois<br>☐Souvent<br>☐ Tous les jours, ou presque tous les jours
11. Taken longer than others to eat a meal?<br>☐Never<br>☐Once or twice<br>☐Sometimes<br>☐Often<br>☐ Everyday or almost everyday	11. Pris plus longtemps que les autres pour manger son repas?<br>☐Jamais<br>☐Une ou deux fois<br>☐Quelques fois<br>☐Souvent<br>☐ Tous les jours, ou presque tous les jours

12. Had difficulty drinking or eating hot or cold foods?<br>☐Never<br>☐Once or twice<br>☐Sometimes<br>☐Often<br>☐ Everyday or almost everyday	12. Eu des difficultés à boire ou à manger de la nourriture chaude ou froide?<br>☐Jamais<br>☐Une ou deux fois<br>☐Quelques fois<br>☐Souvent<br>☐ Tous les jours, ou presque tous les jours
13. Had difficulty eating foods he/she would like to eat?<br>☐Never<br>☐Once or twice<br>☐Sometimes<br>☐Often<br>☐ Everyday or almost everyday	13. Eu des difficultés à manger de la nourriture qu'il aurait aimé manger ?<br>☐Jamais<br>☐Une ou deux fois<br>☐Quelques fois<br>☐Souvent<br>☐ Tous les jours, ou presque tous les jours
During the last 3 months, because of his/her teeth, lips, mouth, or jaws, how often has your child:<br>14. Had diet restricted to certain types of food (e.g. soft food)?<br>☐Never<br>☐Once or twice<br>☐Sometimes<br>☐Often<br>☐ Everyday or almost everyday	*Au cours des trois derniers mois, à cause de ses dents, lèvres, bouche ou mâchoires, combien de fois votre enfant a-t-il :*<br>14. Du limiter son alimentation à certains types d'aliments? (ex : aliments mous)<br>☐Jamais<br>☐Une ou deux fois<br>☐Quelques fois<br>☐Souvent<br>☐ Tous les jours, ou presque tous les jours
SECTION 3: *The following questions ask about the effects that the condition of children's teeth, lips, mouth and jaws may have on their feelings and everyday activities*	SECTION 3: *Les questions suivantes concernent l'impact de l'état des dents, lèvres, bouches ou mâchoires sur leurs ressentis et leurs activités quotidiennes.*
During the last 3 months, because of his/her teeth, lips, mouth or jaws, how often has your child been:	*Au cours des 3 derniers mois, à cause de ses dents, lèvres, de sa bouche ou de ses mâchoires, combien de fois votre enfant a-t'il été ?*
15. Upset?<br>☐Never<br>☐Once or twice<br>☐Sometimes<br>☐Often<br>☐ Everyday or almost everyday	15. Contrarié?<br>☐Jamais<br>☐Une ou deux fois<br>☐Quelques fois<br>☐Souvent<br>☐ Tous les jours, ou presque tous les jours
16. Irritable or frustrated?<br>☐Never<br>☐Once or twice<br>☐Sometimes<br>☐Often<br>☐ Everyday or almost everyday	16. Irritable ou frustré?<br>☐Jamais<br>☐Une ou deux fois<br>☐Quelques fois<br>☐Souvent<br>☐ Tous les jours, ou presque tous les jours
17. Anxious or fearful?<br>☐Never<br>☐Once or twice<br>☐Sometimes	17. Anxieux ou craintif ?<br>☐Jamais<br>☐Une ou deux fois<br>☐Quelques fois

☐Often	☐Souvent
☐ Everyday or almost everyday	☐ Tous les jours, ou presque tous les jours
During the last 3 months, because of his/her teeth, lips, mouth or jaws, how often has your child:	*Au cours des 3 derniers mois, à cause de ses dents, lèvres, de sa bouche ou de ses mâchoires, combien de fois votre enfant a-t'il :*
18. Missed school (e.g. pain, appointments, surgery)?	18. Manqué l'école (par exemple à cause des douleurs, d'un rendez-vous, ou d'une intervention)?
☐Never	☐Jamais
☐Once or twice	☐Une ou deux fois
☐Sometimes	☐Quelques fois
☐Often	☐Souvent
☐ Everyday or almost everyday	☐ Tous les jours, ou presque tous les jours
19. Had a hard time paying attention in school?	19. A eu du mal à être attentif à l'école?
☐Never	☐Jamais
☐Once or twice	☐Une ou deux fois
☐Sometimes	☐Quelques fois
☐Often	☐Souvent
☐ Everyday or almost everyday	☐ Tous les jours, ou presque tous les jours
20. Not wanted to speak or read out loud in class?	20. N'a pas voulu parler ou lire à haute voix en classe?
☐Never	☐Jamais
☐Once or twice	☐Une ou deux fois
☐Sometimes	☐Quelques fois
☐Often	☐Souvent
☐ Everyday or almost everyday	☐ Tous les jours, ou presque tous les jours
21. Not wanted to talk to other children?	21. N'a pas voulu parler avec d'autres enfants?
☐Never	☐Jamais
☐Once or twice	☐Une ou deux fois
☐Sometimes	☐Quelques fois
☐Often	☐Souvent
☐ Everyday or almost everyday	☐ Tous les jours, ou presque tous les jours
22.Avoided smiling or laughing when around other children?	22. A évité de sourire ou de rire en présence d'autres enfants autour ?
☐Never	☐Jamais
☐Once or twice	☐Une ou deux fois
☐Sometimes	☐Quelques fois
☐Often	☐Souvent
☐ Everyday or almost everyday	☐ Tous les jours, ou presque tous les jours
During the last 3 months, because of his/her teeth, lips, mouth or jaws, how often has your child:<br>23. Worried that he/she is not as healthy as other people?	*Au cours des 3 derniers mois, à cause de ses dents, lèvres, de sa bouche ou de ses mâchoires, combien de fois votre enfant a-t'il :*<br>*23.* été inquiet de ne pas être en aussi bonne santé que les autres?
☐Never	☐Jamais
☐Once or twice	☐Une ou deux fois
☐Sometimes	☐Quelques fois
☐Often	☐Souvent

☐ Everyday or almost everyday<br><br>24. Worried that he/she is different than other people?<br>☐Never<br>☐Once or twice<br>☐Sometimes<br>☐Often<br>☐ Everyday or almost everyday<br>25. Worried that he/she is not as good-looking as other people?<br>☐Never<br>☐Once or twice<br>☐Sometimes<br>☐Often<br>☐ Everyday or almost everyday<br><br>26. Acted shy or embarrassed?<br>☐Never<br>☐Once or twice<br>☐Sometimes<br>☐Often<br>☐ Everyday or almost everyday<br><br>27. Been teased or called names by other children?<br>☐Never<br>☐Once or twice<br>☐Sometimes<br>☐Often<br>☐ Everyday or almost everyday<br><br>28. Been left out by other children?<br>☐Never<br>☐Once or twice<br>☐Sometimes<br>☐Often<br>☐ Everyday or almost everyday<br><br>29. Not wanted or been unable to spend time with other children?<br>☐Never<br>☐Once or twice<br>☐Sometimes<br>☐Often<br>☐ Everyday or almost everyday<br><br>30. Not wanted or been unable to participate in activities such as sports,<br>clubs, drama, music, school trips?<br>☐Never<br>☐Once or twice<br>☐Sometimes<br>☐Often<br>☐ Everyday or almost everyday	☐ Tous les jours, ou presque tous les jours<br><br>24. été inquiet d'être différent des autres?<br>☐Jamais<br>☐Une ou deux fois<br>☐Quelques fois<br>☐Souvent<br>☐ Tous les jours, ou presque tous les jours<br>25. été inquiet de ne pas être aussi beau que les autres?<br><br>☐Jamais<br>☐Une ou deux fois<br>☐Quelques fois<br>☐Souvent<br>☐ Tous les jours, ou presque tous les jours<br><br>26. A été timide ou gêné?<br>☐Jamais<br>☐Une ou deux fois<br>☐Quelques fois<br>☐Souvent<br>☐ Tous les jours, ou presque tous les jours<br><br>27. A été embêté ou moqué par d'autres enfants?<br>☐Jamais<br>☐Une ou deux fois<br>☐Quelques fois<br>☐Souvent<br>☐ Tous les jours, ou presque tous les jours<br><br>28. A été mis à l'écart par d'autres enfants ?<br>☐Jamais<br>☐Une ou deux fois<br>☐Quelques fois<br>☐Souvent<br>☐ Tous les jours, ou presque tous les jours<br><br>29. N'a pas voulu ou n'a pas pu passer du temps avec d'autres enfants?<br>☐Jamais<br>☐Une ou deux fois<br>☐Quelques fois<br>☐Souvent<br>☐ Tous les jours, ou presque tous les jours<br><br>30. N'a pas voulu ou n'a pas pu participer à des activités comme du sport, du théâtre, de la musique, des sorties scolaires?<br>☐Jamais<br>☐Une ou deux fois<br>☐Quelques fois<br>☐Souvent<br>☐ Tous les jours, ou presque tous les jours

31. Worried that he/she has fewer friends?<br>☐Never<br>☐Once or twice<br>☐Sometimes<br>☐Often<br>☐ Everyday or almost everyday<br><br>*During the last 3 months, how often has your child been:*<br><br>32. Concerned what other people think about his/her teeth, lips, mouth or jaws?<br><br>☐Never<br>☐Once or twice<br>☐Sometimes<br>☐Often<br>☐ Everyday or almost everyday<br><br>33. Asked questions by other children about his/her teeth, lips, mouth or jaws?<br>☐Never<br>☐Once or twice<br>☐Sometimes<br>☐Often<br>☐Everyday or almost everyday<br><br>SECTION 4 : Child's gender and gender<br>**Your child is:**<br>☐ **Male**<br>☐ **Female**<br>**Your child's age is: ______YEARS**<br>**Questionnaire completed by:**<br>☐ Mother<br>☐ Father<br>☐ Other<br>**Date completed:**<br>______________<br>_______ / _______ / _______<br>DA Y MONTH YEAR	31. Eté inquiet d'avoir moins d'amis que les autres?<br>☐Jamais<br>☐Une ou deux fois<br>☐Quelques fois<br>☐Souvent<br>☐ Tous les jours, ou presque tous les jours<br>*Au cours des 3 derniers mois, combien de fois votre enfant:*<br><br>32. A t-il été interrogé par d'autres enfants au sujet de ses dents, lèvres, bouches ou mâchoires?<br><br>☐Jamais<br>☐Une ou deux fois<br>☐Quelques fois<br>☐Souvent<br>☐ Tous les jours, ou presque tous les jours<br><br>33. S'est il soucié de ce que pensent les autres de ses dents, lèvres, bouches ou mâchoires?<br>☐Jamais<br>☐Une ou deux fois<br>☐Quelques fois<br>☐Souvent<br>☐Tous les jours, ou presque tous les jours<br><br>SECTION 4 : *Age et sexe de votre enfant.*<br>Votre enfant est :<br>☐Garçon<br>☐Fille<br>Votre enfant est âgé de :…………ans<br>Questionnaire complété par:<br>☐Mère<br>☐Père<br>☐Autre<br>Date:<br>----------------<br>...../....../......<br>Jour/Mois/Année

Supplementary data from (1) Razanamihaja N, Boy-Lefèvre ML, Jordan L, Tapiro L, Berdal A, de la Dure-Molla M, et al. Parental-Caregivers Perceptions Questionnaire (P-CPQ): translation and evaluation of psychometric properties of the French version of the questionnaire. BMC Oral Health. 11 2018;18(1):211.
