# Supplementary material for: Orofacial Features, Oral Health-Related Quality of Life, and Exposure to Bullying in Osteogenesis Imperfecta: A Cross-Sectional Study
Source: Children (Basel). 2024 Jul 26;11(8):900. doi: 10.3390/children11080900 (PMC11352506; doi:10.3390/children11080900)
Supplement: Supplementary file 1 [file children-11-00900-s001.zip › File S2. COHIP.pdf]

**COHIP questionnaire from (1)**

1. **El Osta N, Pichot H, Soulier-Peigue D, Hennequin M, Tubert-Jeannin S. Validation of the child oral health impact profile (COHIP) french questionnaire among 12 years-old children in New Caledonia. Health Qual Life Outcomes. 30 oct 2015;13:176.**

**Domain 1: Oral health**

Q1: Had pain in your teeth/toothache
Q2: Been breathing through your mouth or snoring
Q3: Had discoloured teeth or spots on your teeth
Q4: Had crooked teeth or spaces between your teeth

Q5: Had sores/sore spots in or around your mouth
Q6: Had bad breath
Q7: Had bleeding gums
Q8: Had food sticking in or between your teeth
Q9: Had pain or sensitivity in teeth with hot/cold things

Q10: Had dry mouth or lips

**Domain 2: Functional Well-Being**
Q11: Had trouble biting/chewing apple, carrot/firm meat
Q15: Had difficulty eating foods you would like to eat
Q21: Had trouble sleeping
Q26: Had difficulty saying certain words
Q30: People had difficulty understanding what you were saying

Q32: Had difficulty keeping your teeth clean

**Domain 3: Socio-emotional Well-Being**
Q12: Been unhappy or sad
Q16: Felt worried or anxious
Q20: Avoided smiling or laughing with other children

Q27: Felt that you look different

Q33: Been worried about what other people think
Q18: Felt shy or withdrawn
Q25: Been teased, bullied or called names by other children

Q23: Got angry

**Domain 4: School/Environment**
Q13: Missed school
Q19: Had difficulty paying attention in school

Q24: Did not want to speak/read out loud in class

Q36: Did not want to go to school

**Domain 5: Self-image**
Q14: Been reassured or put in trust through
Q31: Felt that you were good looking
Q39: Felt having healthy teeth
Q43: Felt good about himself
Q40: When I am older, I believe that I'll have good teeth

Q41: When I am older, I believe that I will be healthy
