# Supplementary material for: Orofacial Features, Oral Health-Related Quality of Life, and Exposure to Bullying in Osteogenesis Imperfecta: A Cross-Sectional Study
Source: Children (Basel). 2024 Jul 26;11(8):900. doi: 10.3390/children11080900 (PMC11352506; doi:10.3390/children11080900)
Supplement: Supplementary file 1 [file children-11-00900-s001.zip › File S3. bullying questionnaire.pdf]

Appendix S10: Final 26-item (2x13) BCS-A – Ordinal Scale

From (1)1. Thomas HJ, Scott JG, Coates JM, Connor JP. Development and validation of the Bullying and Cyberbullying Scale for Adolescents: A multi-dimensional measurement model. Br J Educ Psychol. mars 2019;89(1):75-94.

Bullying and Cyberbullying Scale for Adolescents (BCS-A-ordinal)

Please read the following information carefully.

A student is being bullied when another student or group of students does one or more of the following:

- Says mean or hurtful things, makes fun of them, calls them names or threatens them.
- Leaves them out of a group or an activity, or won't let them join in, on purpose.
- Hits, kicks or pushes them around.
- Spreads lies or rumours to make others not like them.
- Uses the Internet or mobile phones
  - to send them mean or hurtful messages using words, pictures, or videos.
  - to send other people mean or hurtful messages about them.
  - to spread rumours/lies to make others not like them.
  - to leave a person out or not let them join in, on purpose.

It is bullying when these actions happen again and again, and it is difficult for the person to defend themselves or make it stop happening.

** It is NOT bullying when teasing is done in a friendly and playful way.

** It is NOT bullying if two people who are as strong as each other argue or fight.

Please use this information to help you answer the following questions.

In the past <u>3 months</u>, how many times have you <u>been bullied</u> **"OFFLINE" / FACE-TO-FACE**?

Another student or students…

Punched, hit, kicked, pushed or shoved me, on purpose.

Forced me to do something I did not want to do.

Told me others would not like me if I did not do what they said.

Damaged, hid, or stole my belongings, on purpose.

Called me mean or hurtful names.

Said mean or hurtful things to me.

Left me out of a group or an activity, or did not allow me to join in, on purpose.

Spread lies or rumours about me, to hurt me or make others not like me.

This did not happen to me, Once or twice, Every few weeks, About once a week, Several times a week or more

In the past <u>3 months</u>, how many times have you <u>been bullied</u> **"ONLINE" / ON THE INTERNET or MOBILE PHONES**?

Another student or students…

Called me mean or hurtful names.

Sent or posted, mean or hurtful pictures/videos about me.

Told me others would not like me if I did not do what they said.

Left me out of a group or an activity, or did not allow me to join in, on purpose.

Spread lies or rumours about me, to hurt me or make others not like me.

This did not happen to me, Once or twice, Every few weeks, About once a week, Several times a week or more

In the past <u>3 months</u>, how many times have you <u>bullied</u> another school student "**OFFLINE" / FACE-TO-FACE** – on your own or as part of a group?

Another student or students…

Punched, hit, kicked, pushed or shoved someone, on purpose.

Forced someone to do something they did not want to do.

Told someone that others would not like them if they did not do what I/we said.

Damaged, hid, or stole someone's belongings, on purpose.

Called someone mean or hurtful names.

Said mean or hurtful things to someone.

Left someone out of a group or an activity, or did not allow them to join in, on purpose.

Spread false rumours about a person, to hurt them or make others not like them.

This did not happen to me, Once or twice, Every few weeks, About once a week, Several times a week or more

In the past <u>3 months</u>, how many times have you <u>bullied</u> another school student **"ONLINE" / ON THE INTERNET or MOBILE PHONES** – on your own or as part of a group?

Another student or students…

Called someone mean or hurtful names.

Sent or posted, mean or hurtful pictures/videos about someone.

Told someone that others would not like them if they did not do what I/we said.

Left someone out of a group or an activity, or did not allow them to join in, on purpose.

Spread lies or rumours about someone, to hurt them or make others not like them.

This did not happen to me, Once or twice, Every few weeks, About once a week, Several times a week or more
